# Empathy, personality traits, and emotional management in 2nd and 4th-year dentistry students: a single-center study

**DOI:** 10.1186/s12909-021-03080-1

**Published:** 2022-01-03

**Authors:** Christian Lermen, Willi Wetzel, Vanessa Britz, Jasmina Sterz, Wolf O Bechstein, Teresa Schreckenbach

**Affiliations:** 1 Goethe-University Frankfurt/Main, Department of General, Visceral, Transplantation and Thoracic Surgery, Goethe-University Frankfurt am Main, Frankfurt University Hospital and Clinics, Theodor-Stern-Kai 7, 60596 Frankfurt/Main, Germany; 2 Department of Oral Surgery and Implantology, Goethe-University Frankfurt am Main, Carolinum, Theodor-Stern-Kai 7, 60596 Frankfurt/Main, Germany; 3 Goethe-University Frankfurt am Main, Frankfurt interdisciplinary simulation center FIneST, Medical Faculty, Goethe University, Frankfurt, Germany; 4 Goethe-University Frankfurt am Main, Department of Trauma, Hand and Reconstructive Surgery, University Hospital Frankfurt, Goethe-University Frankfurt am Main, Frankfurt/Main, Germany

**Keywords:** Empathy, Personality inventory, Emotional intelligence, Dental students

## Abstract

**Background:**

Patients fearing dental interventions are at risk of delaying or skipping much-needed treatments. Empathic communication could lead to a higher rate of compliance from patients within this group. Empathy, the big five personality traits, and emotion management abilities are all known to influence the quality of communication between dentists and patients. This study was conducted to analyze whether there is a correlation between these factors in dentistry students.

**Methods:**

Dentistry students in their 2nd and 4th year of study were asked to complete questionnaires assessing empathy, emotion management, and personality traits. Out of a total of 148 eligible participants, 53 students (34%) volunteered to participate. For empathy, the Jefferson Scale of Physician Empathy (students’ version; JSPE-S) and the Interpersonal Reactivity Index (IRI) were used. Personality traits were assessed using the Short Big Five Inventory (BFI-s), and the Situational Test of Emotional Management (STEM) to measure emotional management ability.

**Results:**

Higher scores for emotion management were significantly correlated with the female gender (*p* ≤ 0.005) and with higher scores in openness (*p* ≤ 0.05). Students with higher scores in openness also achieved higher scores on the IRI subscales: Perspective taking (p ≤ 0.05), Fantasy (*p* ≤ 0.01), Empathic concern (p ≤ 0.05), and Personal distress (p ≤ 0.05). For JSPE-S, no correlation with emotion management and personality traits was found.

**Conclusion:**

Empathy and emotion management might not be significantly related in dentistry students. Regarding personality traits, students who scored higher on openness also indicated higher abilities in emotion management. These findings should be taken into consideration when planning communication courses for dentistry students, as it might be possible to independently train empathy and emotion management as part of emotional intelligence.

## Introduction

Face-to-face communication should not be underestimated as part of the doctor-patient relationship [1]. Especially in situations that are associated with fear for a greater number of patients, communication can make the difference [1, 2]. Around 60% of patients in Germany fear dental interventions [3]. Worldwide, between 6 and 21% of dental patients report a severe fear of the dentist [3]. Patients with dental fear are more likely to delay necessary controls and treatments, which can result in the loss of teeth [4, 5]. Patient-centered communication could prevent at least some of the delayed dental treatments [5].

Therefore, the realization that empathic communication makes a decisive contribution to therapy adherence and success is also evident in the new revision of the dental license regulations in Germany [6]. In this revision of the license to practice, which originally dates back to 1955, communication is set for the first time as a learning goal within the framework of dental/medical studies [6]. The recommendation results from the learning objectives of the German National Competence-Based Learning Objectives Catalog of Dentistry (NKLZ), which was introduced in 2015 [7]. The NKLZ describes the graduate profile of dentists from dental/medical school and should serve as an orientation for faculties and dentistry students. It relates to the CanMEDs rolls [8], and defines the dentist as a communicator who is capable of effective patient-centered communication [7].

Empathy is one part of effective patient-centered communication [1]. It is also part of the concept of Emotional Intelligence, which is needed for successful communication [9]. There are multiple models of emotional intelligence, for example, the ability and trait emotional intelligence and mixed models [10]. Most of them understand emotional intelligence as a combination of various components: (1) perceiving emotion (emotion recognition), (2) facilitating thought with emotion, (3) understanding emotion, and (4) managing emotion [11]. Managing emotions does not only mean managing the emotions of other people but also one’s own emotions [9]. High scores on emotion management ability relates to successful communication and the achievement of personal and interpersonal goals [12]. Improving one’s own strategies for emotion management could help to improve empathic communication in doctor-patient relationships [13]. This would be a possible aim for promotion in dentistry students.

For pro-social communications, it was shown that possessing high levels of emotional intelligence is not enough [14]. How emotional intelligence is used in interpersonal communication is dependent on certain personality traits [15]. For example, whether individuals use their emotion recognition ability for prosocial behavior depends strongly on their personality traits [15, 16]. There are different theories for personality traits. One of the most often used and well-established theories is the Five-Factor model, also known as the Big Five Inventory, defining five broad dimensions of personality traits: (1) openness, (2) conscientiousness, (3) extraversion, (4) agreeableness, and (5) neuroticism [17].

This raises the question of whether there is a correlation between emotion management, personality traits and empathy. Collecting data for interrelationship areas of emotional intelligence is important to understand the complex interplay of individual emotional abilities. For creating effective teaching methods for patient-centered communication, it is important to understand this relationship. So far, no studies analyzing emotion management and its relationship with student’s personality or empathy have been conducted with dentistry students.

This study attempted to answer the questions of whether 1) self-assessed empathy and emotion management abilities differ between 2nd and 4th year dentistry students, and 2) whether there is a correlation between empathy, personality traits, and emotion management abilities.

## Materials and methods

### Study protocol

This study was conducted in accordance with the Helsinki Declaration (Ethical Principles for Medical Research Involving Human Subjects), and the study was performed with the permission of the Ethics Committee of the Goethe University, Frankfurt, Germany (IRB No. 19–225). All dental students enrolled in the second (*n* = 86) and fourth year (*n* = 62) at the University of Frankfurt/Main were asked to participate in this study. Participation in the study was voluntary and took place after written informed consent. The questionnaires were completed anonymously so that no conclusions could be drawn about the student involved.

### Self-assessment of empathy

For empathy assessment two self-assessment questionnaires were used. The Jefferson Scale of Physician Empathy Students version (JSPE-S) and the Interpersonal Reactivity Index (IRI) are widely known and used, which offers an option for international comparison [18, 19]. Self-assessment was used to enable a better comparison of the assessed level of empathy between the students of the different academic years. Students in the 4th year of study have already gained experience with patients compared to those in the 2nd year, which could falsify a patient-centered empathy assessment, as it is used in the Consultation and Relational Empathy (CARE) Measure [20].

#### Jefferson scale physician empathy students version (JSPE-S)

The German version of the Jefferson Scale Physician Empathy Students Version (JSPE-S) was used to measure the students’ subjectively perceived empathy relevance in medicine [21]. The JSPE-S is a 20-item-questionnaire [18]. Respondents indicate their level of agreement on a 7-point Likert scale [1 (strongly disagree)–7 (strongly agree)]. The test assesses the participants’ level of empathy based on three subcategories: Perspective Taking (range, 10 to 70 points), Compassionate Care (range, 8 to 56 points), and walking in patients’ shoes (range, 2 to 14 points) [18].

#### Interpersonal reactivity index (IRI)

The German version of the Interpersonal Reactivity Index (IRI) as a self-report, comprising 28-items answered on a 5-point Likert scale, was used to conduct a multidimensional assessment of empathy [22]. It is divided into four subscales: 1. Perspective Taking, 2. Fantasy, 3. Empathic Concern, and 4. Personal Distress [19]. Each participant can receive between 0 to 28 points on each subscale.

### Personality traits - BIG-five-inventory short version (BFI-K)

Personality traits were determined using the German version of the Big Five Inventory short version (BFI-K) questionnaire, which was established in 2005 as a short version of the BFI [23]. The items were rated on a 5-point Likert scale ranging from 1 = strongly disagree.

to 5 = strongly agree. It consists of 21 items assessing participants level of extraversion (range, 4 to 20 points), openness to experience (range, 5 to 25 points), conscientiousness (range, 4 to 20 points), agreeableness (range, 4 to 20 points), and neuroticism (range, 4 to 20 points) [23].

### Situational test of emotional management (STEM)

Emotion management abilities were measured using the Situational Test of Emotion Management (STEM) [24]. This consists of 44 multiple-choice items. Each item consists of a description of an emotional situation as well as four possible reactions [24]. The participants had to decide which reaction would be the most suitable. There are no correct answers in the conventional sense. However, answers were ranked by experts according to suitability. Students received one point for choosing the answer most preferred by experts.

### Statistics

All statistical analyses were performed using International Business Machines Corporation (IBM) Statistical Package for the Social Sciences (SPSS) for Windows (version 25.0; IBM, Chicago, IL, USA). Cronbach’s alpha was used to evaluate the reliability of the questionnaires. For analyzing the difference between the students, Mann-Whitney U test as non-parametric test for not equally distributed parameters was used. The associations between emotional management, empathy, and each personality trait were assessed using Pearson’s Correlation Coefficient. All correlations were two-tailed, and a *p*-value < 0.05 was considered statistically significant.

## Results

### Participants

Out of the total of 148 eligible participants, 53 students (34%) volunteered to participate. Thirty questionnaires were received from the second-year students and 23 from the fourth-year students. Thirty-four students were female, and 16 were male. The median age was 24 years (SD, 3.8), with a range from 19 to 32 years.

Of all students, 33 (62.3%) had no previous vocational training before enrollment at dental/medical school. There were no differences between the second- and fourth-year students for scores in JSPE-S, IRI, BFI-K, and STEM (Table 1).

**Table 1:** Demographic data of participants and descriptive analyses of JSPE-S, IRI, BFI-K, and STEM

	Students 2nd year N (%)	Students 4th year N (%)	P-Value
Gender			0.973
female	21 (70.0)	16 (69.6)	
male	9 (30.0)	7 (30.4)	
Vocational Training (before enrollment at dentistryl school)			0.161
nurse	0 (0.0)	1 (4.3)	
dental assistant	1 (3.3)	0 (0.0)	
dental technician	3 (10.0)	3 (13.0)	
others	10 (33.3)	2 (8.7)	
	Students 2nd year Mean (SD)	Students 4th year Mean (SD)	P-Value
Age (in years)	23.8 (4.5)	23.8 (2.7)	0.995
JSPE-S			
Perspective Taking	54.9 (8.02)	56.6 (6.7)	0.425
Compassionate Care	41.4 (7.3)	42.7 (5.5)	0.492
Walking in Patients’ Shoes	9.10 (2.8)	7.8 (2.5)	0.092
IRI			
Perspective Taking	19.2 (4.2)	18.0 (3.4)	0.306
Fantasy Scale	18.2 (6.2)	17.8 (5.4)	0.835
Empathic concern scale	21.6 (4.0)	19.4 (4.8)	0.073
Personal distress Scale	9.2 (4.5)	9.0 (4.4)	0.921
BFI-K			
Agreeableness	12.0 (3.3)	12.4 (3.0)	0.597
Extraversion	15.9 (3.5)	14.3 (2.9)	0.079
Openness	20.7 (3.1)	21.1 (2.8)	0.636
Conscientiousness	15.2 (2.8)	16.2 (2.1)	0.165
Neuroticism	11.7 (3.7)	10.9 (3.3)	0.429
STEM	31.6 (5.4)	32.4 (4.0)	0.569

### Descriptive analysis of JSPE-S, BFI-K, and STEM

For the JSPE-S subscales, the following mean scores were achieved: Perspective Taking, 55.6 (SD 7.5); Compassionate Care, 42.0 (SD 6.6); Walking in Patients’ Shoes, 8.6 (SD 2.7). Cronbach’s Alpha for the subscales were: “Perspective Taking”: 0.7; “Compassionate Care”: 0.6; “Walking in Patients’ Shoes”: 0.7.

For IRI subscales, the students showed the following mean scores: Perspective Taking, 18.7 (SD, 3.9), Fantasy, 18.0 (SD, 5.8), Empathic Concern, 20.7 (SD, 4.4), and Personal Distress, 9.1 (SD, 4.4). Cronbach’s Alpha for the subscales were: “Perspective Taking”: 0.7; “Fantasy”: 0.8; “Empathic Concern”: 0.8; and “Personal distress”: 0.7.

The results of the BFI-K were: Agreeableness, 12.2 (SD, 3.2), Extraversion, 15.2 (SD 3.3), Openness, 20.9 (SD, 2.9), Conscientiousness, 15.7 (SD, 2.5), and Neuroticism, 11.4 (SD, 3.6). Internal consistency of extraversion (four items) was α = 0.8, openness α = 0.6, agreeableness α = 0.7; conscientiousness α = 0.6; and neuroticism α = 0.8.

For STEM, the students reached a mean score of 31.9 (SD, 4.8). Weighted to the expert results, students achieved 72.4% of the possible best answers. Internal consistency for STEM was α = 0.7.

### Correlation between self-assessed empathy, personality traits, and emotional management abilities

Students scoring high in openness in BFI-K achieved statistically significantly higher scores on all IRI subscales: Perspective Taking (*p* ≤ 0.05), Fantasy (*p* ≤ 0.01), Empathic Concern (p ≤ 0.05), and Personal Distress (p ≤ 0.05). Students who scored highly in agreeableness achieved significantly higher scores on the Empathic Concern scale (p ≤ 0.05) and the Personal distress scale (*p* < 0.05). A conscientious personality was associated with significantly lower scores on the IRI subscales Empathic concern (p ≤ 0.05) and Personal distress (p ≤ 0.05). Neuroticism showed a significant correlation with IRI subscales Fantasy (p ≤ 0.05), Empathic concern (p ≤ 0.01), and Personal distress (*p* = 0.01).

High scores on the STEM questionnaire were significantly correlated with female gender (p < 0.05), higher scores on the IRI-Fantasy subscale (p < 0.05), IRI-Personal distress scale (*p* < 0.001), and higher scores in openness (p < 0.05). The complete results are shown in Table 2.

**Table 2:** Pearson-Correlation between demographic data, subscales of JSPE-S and IRI, BFI-K, and STEM (showing the Pearson correlation factor)

		*M*	*SD*	1	2	3	4	5	6	7	8	9	10	11	12	13	14	15
1	Age	23.8	3.8															
2	Gender	0.7	0.5	−0.118														
3	Year of study	1.4	0.5	−0.001	−0.005													
4	JSPE-S: Perspective Taking	55.6	7.5	−0.065	0.122	0.112												
5	JSPE-S: Compassionate Care	42.0	6.6	0.255	0.289*	0.096	0.475**											
6	JSPE-S: Walking in Patients Sh.	8.6	2.7	0.133	0.255	−0.234	0.162	0.179										
7	IRI: Perspective Taking	18.7	3.9	−0.039	0.178	−0.143	0.113	0.086	0.177									
8	IRI: Fantasy Scale	18.0	5.8	−0.176	0.372**	−0.029	0.322*	0.380**	0.140	0.317*								
9	IRI: Empathic concern scale	20.7	4.4	0.007	0.485**	−0.248	0.184	0.297*	0.307*	0.217	0.501**							
10	IRI: Personal distress Scale	9.1	4.4	−0.250	0.422**	− 0.014	0.174	0.111	−0.031	− 0.081	0.327*	0.404*						
11	Agreeableness	12.2	3.2	−0.137	0.272*	0.074	0.188	0.120	0.085	−0.002	0.215	0.288*	0.335*					
12	Extraversion	15.2	3.3	−0.148	0.059	− 0.243	0.124	0.082	0.128	0.040	0.084	0.035	−0.065	−0.143				
13	Openness	20.9	2.9	−0.074	0.326*	0.067	−0.060	0.136	0.106	0.283*	0.569**	0.283*	0.292*	0.065	0.058			
14	Conscientiousness	15.7	2.5	0.068	0.124	0.194	−0.158	0.084	0.055	0.205	0.017	−0.284*	−0.307*	− 0.074	0.138	0.165		
15	Neuroticism	11.4	3.6	−0.072	0.453**	−0.111	0.187	0.209	0.067	0.010	0.333*	0.379**	0.581**	0.135	−0.132	0.197	−0.193	
16	STEM	31.9	4.8	−0.149	0.438**	0.082	0.095	0.160	0.218	0.178	0.285*	0.208	0.317**	0.006	0.195	0.301*	0.230	0.270

*N* = 53; * p < =0.05; ** p < =0.01

*M* mean; *SD* Standard Deviation

## Discussion

Emotional intelligence and empathy are part of patient-centered communication and are known to differ between individuals, depending on personality traits, amongst other things [25, 26]. For creating effective teaching methods for patient-centered communication, it is important to understand this relationship.

Thus, this study was conducted to analyze the correlation between self-assessed empathy, personality traits, and emotion management abilities as one part of emotional intelligence in dentistry students. This demonstrates that female students and students with a more open personality scored the highest for self-assessed empathy and showed the best emotion management abilities.

One of the most frequently examined factors influencing empathy and emotional intelligence is gender. Many studies have shown that women score higher in self-assessed empathic behavior and emotional intelligence [27–29]. One reason for this could be that women often express more emotions and have shown higher skills for effective interpersonal relationships and communication [18, 29]. However, some studies have suggested that this self-assessment could be falsified by socially prescribed role models [30]. Fisher et al. could show that men rated lower in self-reported emotional intelligence but showed the same perception for emotions as women [28]. These statements are supported by studies which have shown that when empathy was assessed by patients and simulated patients, gender had no influence [25]. Other authors suspect not necessarily a difference between men and women for global emotional intelligence but more for single facets of emotional intelligence [29, 31]. Women were shown to score higher for impulse control and relationship, differing from men who showed better stress management [31].

These results can be further extended to the question of what influence has personality on empathy. Studies with self-assessed empathy skills showed individuals with a more agreeable and open personality rated higher for empathy than individuals with other personality traits [32, 33].

In addition to these personality types, emotional intelligence also counts as an influencing factor on empathy. Among other things, emotional intelligence includes the ability to understand emotions, emotional experience, and the ability to manage emotions [9].

The concept of the differences between two main areas of emotional intelligence, trait and ability emotional intelligence, was discussed in previous studies [10]. Emotional intelligence ability is seen as the part of emotional intelligence which can be taught [34]. This part of emotional intelligence could be relevant for the development of teaching concepts for patient-centered communication in dentistry students [35]. In contrast, trait emotional intelligence was shown to be part of individual personality and correlates with the five-factor model of personality [36], in contrast to emotional intelligence ability. The present study found a significant correlation of emotion management abilities as part of emotional intelligence with students’ expression of openness as a personality trait but not with the other four personality traits equal with other studies [37, 38].

For measuring empathy different approaches are used. Some authors use self-assessment questionnaires, similar to this study [27, 33]. However, self-assessment questionnaires could lead to the problem of socially desirable answers [30]. Therefore, other authors had the empathic behavior of students assessed by patients, simulated patients, or teachers [25]. For external assessment the CARE questionnaire can be used. The CARE Scala consists of 10 items, which provide a quick and uncomplicated assessment of empathic behavior [20]. It was developed by Mercer et al. and can be applied to many medical professions [20]. Barbar et al. used the CARE Scale in their study with dentistry students [39]. Second year students achieved higher scores on the CARE Scale than fourth year students. The authors were also able to show a positive correlation between self-assessed empathy and the CARE scale [39].

The decline in empathy, which was shown in that study [39], is also known from medical students [26, 40]. Reasons for this could be an increase in professional behavior, as well as stress, workload, and psychological fatigue [41–43]. Psychological health and well-being of individuals also have an influence on emotion management abilities [24, 44]. Individuals suffering from anxiety and depression show lesser abilities of emotion management than individuals who are mentally healthy [24].

Many studies on empathy use one of the questionnaires mentioned above, which enables comparability between the studies. This reveals the cultural background of the students as another factor influencing empathy and emotional management [25, 45]. Chatterjee at al. used the JSPE-S for Indian medical students and found a lower empathy level compared to studies from other countries [27]. The authors suspect a connection with a more paternalistic doctor-patient relationship model compared to other countries [27].

Since empathy and emotion management are important for a successful doctor-patient relationship, targeted training would be desirable. A 6-year longitudinal study by Kataoka et al. was able to show a positive influence of communication training on empathy of medical students [46]. Daryazadeh et al. used a narrative approach to train self-reflection and empathy and were able to show an increase in both after a session of only two hours [47]. Other studies with a wide variety of training approaches have also shown an improvement in the doctor/student-patient relationship [48].

### Implication for further research and training

A focus of further studies should be on creating courses for patient-centered communication, whose structures are based on the individual skills of the students. For example, students with low scores in emotion management could be taught in stress management abilities, while courses in communication could be offered for students with more introverted or neurotic personality traits.

The results of this study should also be checked in a larger cohort. Furthermore, the connection between empathy, emotional management and the cultural background of the students should be analyzed in more detail. It would be important to find out whether the family’s cultural background or the cultural area of the place of study have an influence.

In order to find out more about the influence of personal distress, it would be helpful to take a closer look using a questionnaire with more detailed questions about personal distress than the IRI. Future research should also include global emotional intelligence measurement and analyses of students’ psychological well-being and depression levels.

### Limitations

This study has a few limitations. First, this investigation focused on students at two different points in dentistry training. Thus, this study might not reflect the performance of students in earlier or later years of dentistry school as it analyses students at the beginning and in the middle of their education to get an overlook about emotion management abilities during dentistry education.

Using self-assessment forms to estimate personality dimensions might have resulted in biased data. Studies have shown that individuals tend to answer in socially acceptable dimensions, which could lead to false data [30]. On the other hand, filling in the questionnaires might result in self-reflection on the part of the participants, which could lead to more self-confidence in communication with patients. For emotion management, only the ability, and not trait emotional intelligence or global emotional intelligence was measured, which only gives a limited view on emotional intelligence.

Only one-third of both student years completed the questionnaire, which resulted in a relatively small number of participants, causing a possible influence on the quantitative analyzes. A higher number of participants may have led to a different, and significant result.

Despite these limitations, this study also has some strengths. To our knowledge, this is the first study analyzing the correlation between empathy, personality traits, and emotion management abilities in dentistry students. The questionnaires used in this study are used internationally and have been evaluated in various studies, and the study design is transferable to other institutions.

## Conclusion

The present study investigated the correlation between self-assessed empathy, personality traits and emotion management abilities in 2nd- and 4th-year dentistry students. No differences between the two years of study could be found. Higher emotional management abilities were correlated with the female gender and a more open personality. Emotional management ability correlated with the IRI subscales “Fantasy” and “Personal Distress”. However, there was no correlation between any subscale of the JSPE or the other subscales of the IRI measuring self-assessed empathy. These findings should be kept in mind when planning communication courses for dentistry students. For example, empathy and emotion management as parts of emotional intelligence might be independently trainable.

## Data Availability

The datasets used and analyzed during the current study are available from the corresponding author on reasonable request.

## Abbreviations

BFI-s: Short Big Five Inventory
IRI: Interpersonal Reactivity Index
JSPE-S: Jefferson Scale of Physician Empathy
NKLZ: German National Competence-Based Learning Objectives Catalog of Dentistry
SD: Standard Deviation
STEM: Situational Test of Emotional Management
